# The electrochemical corrosion behaviour of compacted (Bi, Pb)-2223 superconductors in aqueous solutions

**DOI:** 10.1038/s41598-022-22663-6

**Published:** 2022-10-26

**Authors:** Ashraf M. Abdel-Gaber, Ahmad Najem, Ramadan Awad

**Affiliations:** 1 Department of Chemistry, Faculty of Science, Alexandria University, P.O. Box 426, Alexandria, 21321 Egypt; 2 Department of Physics, Faculty of Science, Beirut Arab University, Beirut, Lebanon; 3 Department of Physics, Faculty of Science, Alexandria University, P.O. Box 426, Alexandria, 21321 Egypt

**Keywords:** Chemical engineering, Electrochemistry

## Abstract

The corrosion behaviour of (Bi, Pb)-2223 samples compacted at 0.3–1.9 GPa in 0.5 M of HCl, NaCl, and NaOH solutions at 30 °C was investigated using potentiodynamic polarization curves measurements and electrochemical impedance spectroscopy (EIS) technique as well as scanning electron microscopy (SEM) and energy dispersive X-ray emission spectroscopy (EDX). Polarization results showed that the increase in compaction decreases both cathodic hydrogen evolution or oxygen reduction and anodic (BiPb)-2223 superconductor dissolution in 0.5 M HCl, and 0.5 M NaOH. On the other hand, compaction mainly affects the anodic part of the polarization curves of (Bi, Pb)-2223 in 0.5 M NaCl solution. EIS measurements revealed that the highest protection of the superconductors was achieved in 0.5 M NaCl, while the lowest degree of protection was observed in 0.5 M HCl. SEM images show a random plate-like morphology fitted with the marker of (Bi, Pb)-2223 material. The compacted sample at 1.9 GPa indicates deformation of the grains and the formation of a micro-crack. The corrosion mechanism of the superconductor at different pH values was also discussed.

## Introduction

For technical experts, superconductors are extremely important due to their unique zero resistivity property. Superconductors have found widespread applications in various fields of science and technology. Recently, they have been used in a variety of applications, including telecommunications, medicine, transportation, defense, space exploration, and power transmission^1–3^. Bi-based superconductors with the Bi-based superconductors with the general formula Bi_2_Sr_2_Ca_*n*-1_Cu_*n*_O_2*n*+4+*δ*_ (*n* = 1, 2, 3) contain three phases. Bi-2223 is the most significant phase, which has a high superconducting transition temperature of 110 K^4^. Bismuth is usually a by-product produced during the production of lead and is often found in the smelting slag of lead anode mud^5^. The partial substitution of Bi^3+^ ions with Pb^2+^ ions improve the structural stability and promotes the formation of the Bi-2223 phase^6^. Such superconducting materials are important for practical applications. For instance, Oyama et al*.* have developed a prototype electric car equipped with a bismuth-based superconducting engine to examine the capabilities and drawbacks of such superconductors^7^. In addition, superconductors have recently been utilized in the design of marine engines to improve their durability and maneuverability^1^. One important factor to consider in achieving such a goal is the rate of corrosion of these materials when placed in media with different pH. Several studies^8–11^ have been conducted to investigate the electrochemical and corrosion behavior of Bi-Pb superconductors in various environments. Mun et al*.*^12^ performed studies on the effect of the uniaxial compacting pressure on the superconducting properties of the Bi-2223 samples. The porosity and volume of the samples were found to decrease with increasing compaction. The structural and transport measurements showed that the intragranular properties of these samples were very similar. The SEM indicated that the compaction and heat treatment were enough to produce a homogenous material. According to Özkurt et al.^13^, the ceramic nature of Bi-2223 high-temperature superconductors limit their practical application: they are very fragile, extremely anisotropic, have a low critical current density at high temperatures, and are difficult to manufacture in a single phase. Chemical storage or removal may depend on a combination of physical properties, such as porosity and surface area, as well as chemical properties, such as surface reactions^14,15^. Given the broad range of chemicals that may be encountered, including highly acidic/acid-forming gases as well as neutral and alkaline chemicals, materials must be developed with certain functionalities capable of retarding the corrosion of different classes of chemicals.

The novelty of the present work arises from studying the effects of compaction and the pH of the surrounding media on the rate of corrosion of the (Bi, Pb)-2223 superconductor.

## Experimental procedures

Measurements of electrochemical impedance (EIS) and potentiodynamic polarization curves were performed using ACM 631 Instruments (UK). For EIS measurements, the frequency range was 0.01 to 3 × 10^4^ Hz with an amplitude of ± 10 mV around the rest potential. An electrochemical cell of a three-electrode mode was employed; the counter and reference electrodes were platinum sheet and saturated calomel electrodes (SCE). The working electrode was made from superconductor specimens with the general formula Bi_1.6_Pb_0.4_Sr_2_Ca_2_Cu_3_O_10-δ_ (Bi, Pb)2223. The preparation of the superconductor was carried out by the standard solid-state reaction technique. Bi_2_O_3_, PbO, PbO_2_, SrCO_3_, CaCO_3_, and CuO powders of high purity were used. The starting powders were mixed in stoichiometric amounts and milled in an agate mortar for one hour. The final grey mixture was calcined for 48 h at 820 °C in a GallenKamp box furnace with intermediate grinding and sieving processes. AMSLER hydraulic press of 300 tons capability was used to compress the calcined powder into cylinder shape at different pressures (0.3, 0.7, 1.0, 1.4, and 1.9 GPa)^16^. The working electrodes were insulated with epoxy resin, leaving only one surface uncovered, with an exposed area of 0.36 cm^2^. The working electrode was left in the test solution (0.5 M HCl, 0.5 M NaCl, or 0.5 M NaOH) for 20 min to acquire the steady-state open circuit potential prior to electrochemical measurements. In the potential range of ± 250 mV, measurements of the polarization curve around the rest potential have been obtained at a scan rate of 30 mV/min. All the experiments were carried out at 30 ± 0.1 °C. For sample morphology, a Scanning Electron microscope (SEM- AIS2300C) was used at a resolution of 20 kV × 4 k. The elemental composition of the prepared samples was analyzed by using an energy dispersive X-ray spectroscopy (EDX) detector-type SDD Apollo X.

## Results and discussion

### SEM and EDX

**Figure 1:**
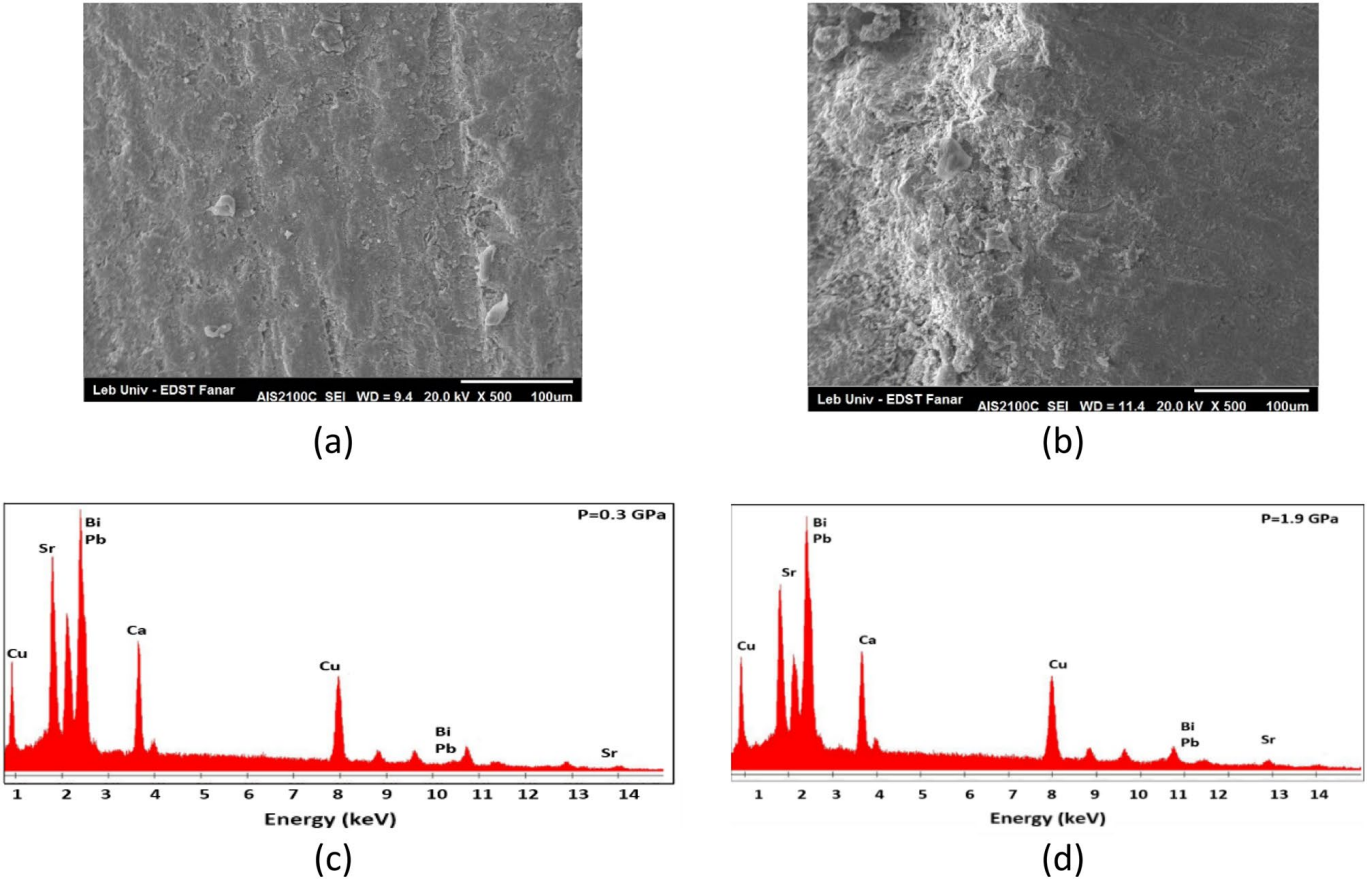
SEM (**a** and **b**) and EDX analysis (**c** and **d**) of (Bi, Pb)-2223 superconductor compacted at P = 0.3 and 1.9 GPa, respectively.

SEM was used to examine the surface morphology, defects, grain size and porosity of the prepared samples. Whereas the EDX technique was used to identify the elemental composition of materials. The SEM and EDX of (Bi, Pb)-2223 samples (P = 0.3 and 1.9 GPa) are shown in Fig. 1a–d. The SEM micrographs shown in Fig. 1a reveal a random plate-like morphology, which is fitted with the fingerprint of (Bi, Pb)-2223 material^17^. As seen in Fig. 1b, upon compaction of the sample to 1.9 GPa, it exhibits deformation of the plate-like morphology and the formation of micro-cracks and defects. These observations are mainly attributed to the effect of compaction on the samples. The EDX analysis for the samples Bi_1.8_ Pb_0.4_ Sr_2_ Ca_2_ Cu_3_ O_10+δ_ is shown in Fig. 1c,d. The peak positions of O, (Bi + Pb), Sr, Ca, and Cu do not change as pressure increases. Bi and Pb peak positions overlap at energies of 2.4 and 10.85 keV, respectively. This is most likely due to the smaller difference in atomic number (*Z*) between the two elements (*Z*_Bi_-*Z*_Pb_ = 1).

The average real compositions of O, Bi + pb, Sr, Ca, and Cu in atomic percentages taken at different regions are shown in Table 1, as well as the ratio of (Bi + Pb)/Cu for all prepared samples. These compositions are unaffected by increasing pressure.

**Table 1:** EDX analysis of (Bi, Pb)-2223 superconductor compacted at different pressure.

P (GPa)	O At %	(Bi, Pb) At %	Sr At %	Ca At %	Cu At %	(Bi, Pb)/Cu
0.3	51.5	10.8	12.0	10.36	15.34	0.70
0.7	51.89	10.9	11.57	10.2	15.44	0.71
1	51.70	11.2	11.19	10.4	15.51	0.72
1.4	52.00	10.85	10.92	10.3	15.93	0.68
1.9	52.03	10.96	10.98	10.5	15.53	0.71

All samples have a (Bi + Pb)/Cu ratio less than one, indicating the formation of the superconducting phase (Bi,Pb)-2223. Furthermore, the sample prepared at 1.4 GPa has the lowest value, which is nearly equivalent to the best value obtained for a single phase = 0.66.

### Porosity

The porosity of (Bi, Pb)-2223 superconductor electrodes compacted at different pressure were determined using the equation (p = [1 − ρ/ρ_th_] × 100) where ρ is the experimental density and the ρ_th_ is theoretical density of Bi, Pb superconductor phase (ρ_th_ = 6.3 g*/*cm^3^)^17^. It is clear from Table 2 that increasing compaction up to 1.4 GPa decreases the porosity of the superconductor’s electrodes which is in a good accordance with the conclusion reported previously that indicated that the pressed materials show reduced porosity after pressing^14,15^. However, no change in porosity was observed after 1.4 GPa compaction, which could be attributed to deformation of the plate-like morphology as shown in the SEM micrograph.

**Table 2:** The porosity of of (Bi, Pb)-2223 superconductor electrodes manufactured at different pressure.

Pressure (GPa)	0.3	0.7	1.0	1.4	1.9
%Porosity	27	23	20	16	16

### Potentiodynamic polarization curves measurements

**Figure 2:**
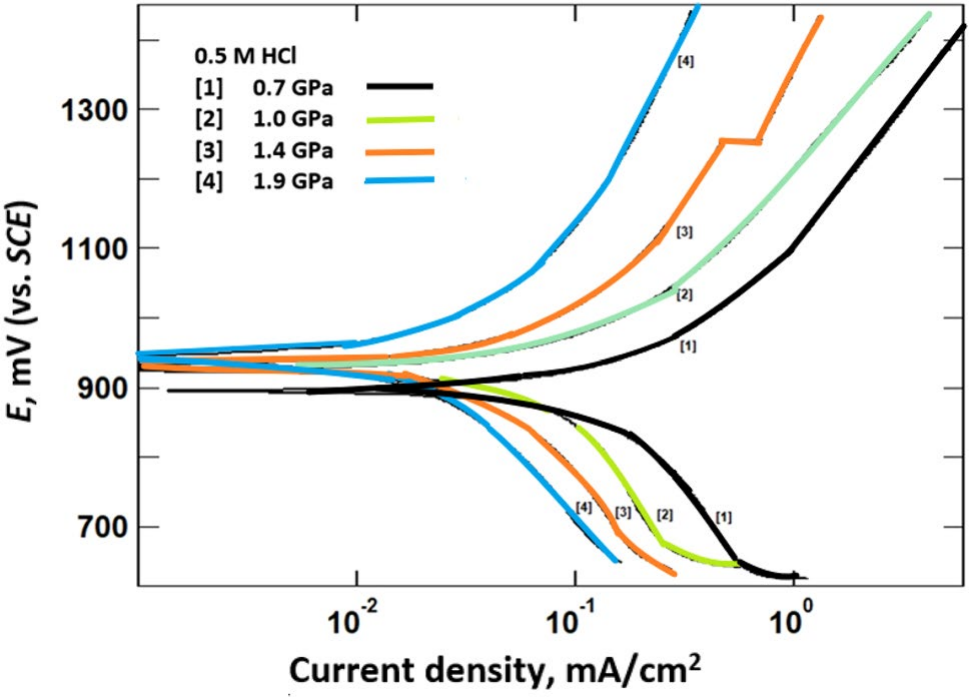
Potentiodynamic polarization curves of (Bi, Pb)-2223 superconductor electrodes compacted at different pressure in 0.5 M HCl.

**Figure 3:**
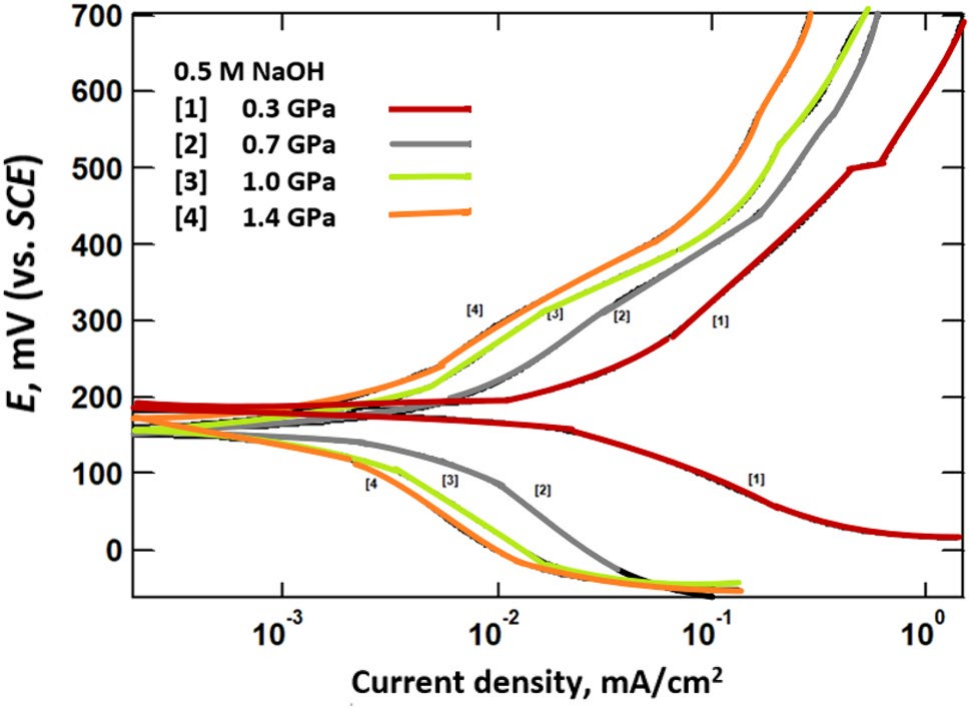
Potentiodynamic polarization curves of (Bi, Pb)-2223 superconductor electrodes compacted at different pressure in 0.5 M NaOH.

**Figure 4:**
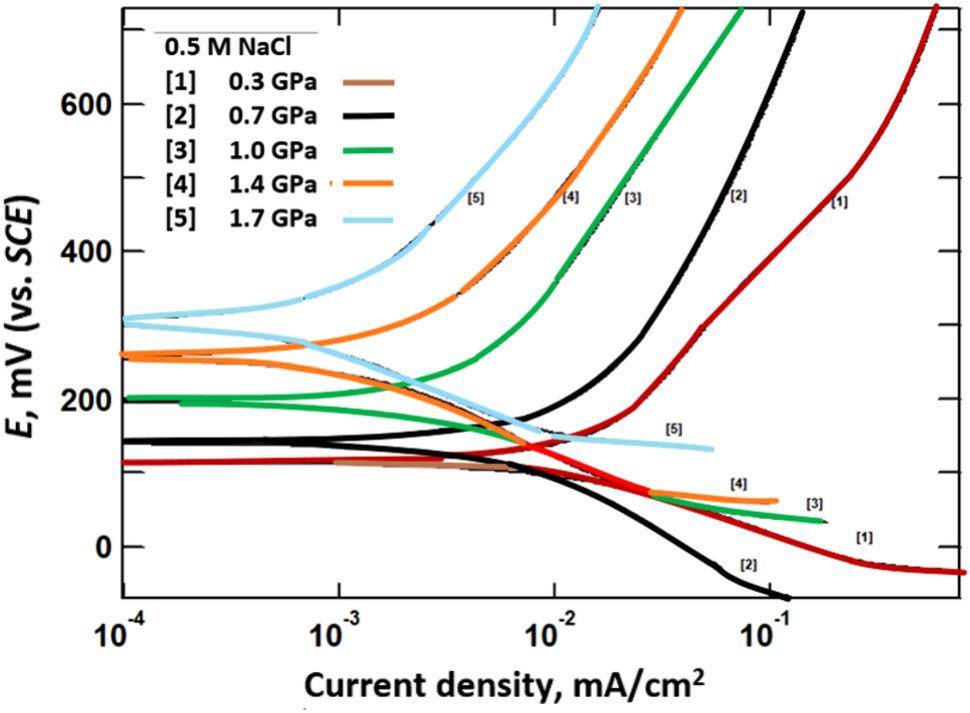
Potentiodynamic polarization curves of (Bi, Pb)-2223 superconductor electrodes compacted at different pressure in 0.5 M NaCl.

Figures 2, 3, 4 show the potentiodynamic polarization curves of (Bi, Pb)-2223 superconductor electrodes compacted at different pressures in 0.5 M HCl, 0.5 M NaOH, and 0.5 M NaCl, respectively. These figures depict a few selected curves. Further data will be shown in Table 3. Figures 2 and 3 show that increasing the pressure inhibits the anodic dissolution of the superconductor and cathodic hydrogen evolution or cathodic oxygen reduction for HCl and NaOH, respectively. On the other hand, the polarization curves of the (Bi, Pb)-2223 superconductor electrode in 0.5 M NaCl, Fig. 4, indicate that pressure predominantly affects the andic portion of the polarization curves. This indicates that the mechanism of dissolution of the semiconductors depends on the solution pH. This will be covered in the section of corrosion mechanism. The electrochemical polarization parameters obtained from the analysis of the curves are shown in Table 3.

**Table 3:** The electrochemical polarization parameters of (Bi, Pb)-2223 superconductor compacted at different pressure in 0.5 M HCl, 0.5 M NaOH and 0.5 M NaCl.

	Pressure, GPa	*E*_corr_ *vs SCE*, mV	*β*_a_	*β*_c_	*i*_corr_, mA/cm^2^
			mV/decade		
0.5 M HCl	0.3	745	390	265	0.2120
	0.7	841	377	425	0.1964
	1.0	873	344	520	0.1017
	1.4	876	420	444	0.0619
	1.9	879	499	333	0.0319
0.5 M NaOH	0.3	174	244	128	0.0238
	0.7	155	183	204	0.0045
	1.0	150	168	182	0.0019
	1.4	159	168	194	0.0014
	1.9	156	159	133	0.0014
0.5 M NaCl	0.3	112	335	112	0.0138
	0.7	110	452	183	0.0101
	1.0	182	430	142	0.0042
	1.4	231	362	166	0.0021
	1.9	277	308	120	0.0008

The superconductor has a higher corrosion potential (*E*_corr_) in 0.5 M HCl than in 0.5 M NaOH or 0.5 M NaCl. This higher positive value reflects the high tendency of the superconductor to corrode in 0.5 M HCl than in the other tested corrosive media. The corrosion current density (*i*_corr_) was obtained from the intersection of Tafel lines (the linear portion that is shown only above ± 50 mV of the corrosion potential, *E*_corr_). The tabulated data clearly shows that increasing pressure decreases the corrosion current density. The decrease in the *i*_corr_ with compaction may be attributed to decreasing porosity of Bi, Pb superconductor that retard the diffusion of the aggressive ions to the electrodes^18^. The values of *i*_corr_ are highest for 0.5 M HCl. The corrosion potential obtained for the superconductor in HCl is shifted to more positive values than that for NaCl and NaOH solutions.

According to the potential-pH diagram of the Pb/Ag/Bi-H_2_O system drawn by Xing et al.^11^ to assess the possibilities of corrosion of metals. The corrosion potentials of (Bi, Pb)-2223 superconductor in 0.5 M HCl indicates the dissolution of lead as Pb^2+^ and bismuth as Bi^3+^. Whereas in NaOH solution, corrosion products of Pb(OH)_2_ and BiOOH are formed. On the other hand, the corrosion products in NaCl, are Pb(OH)_2_ and BiOCl. Therefore, the shift in *E*_corr_ with pH may be due to formation of different ionic species during dissolution of the superconductor in 0.5 M NaCl, NaOH and HCl.

### Electrochemical impedance spectroscopy (EIS) measurements

**Figure 5:**
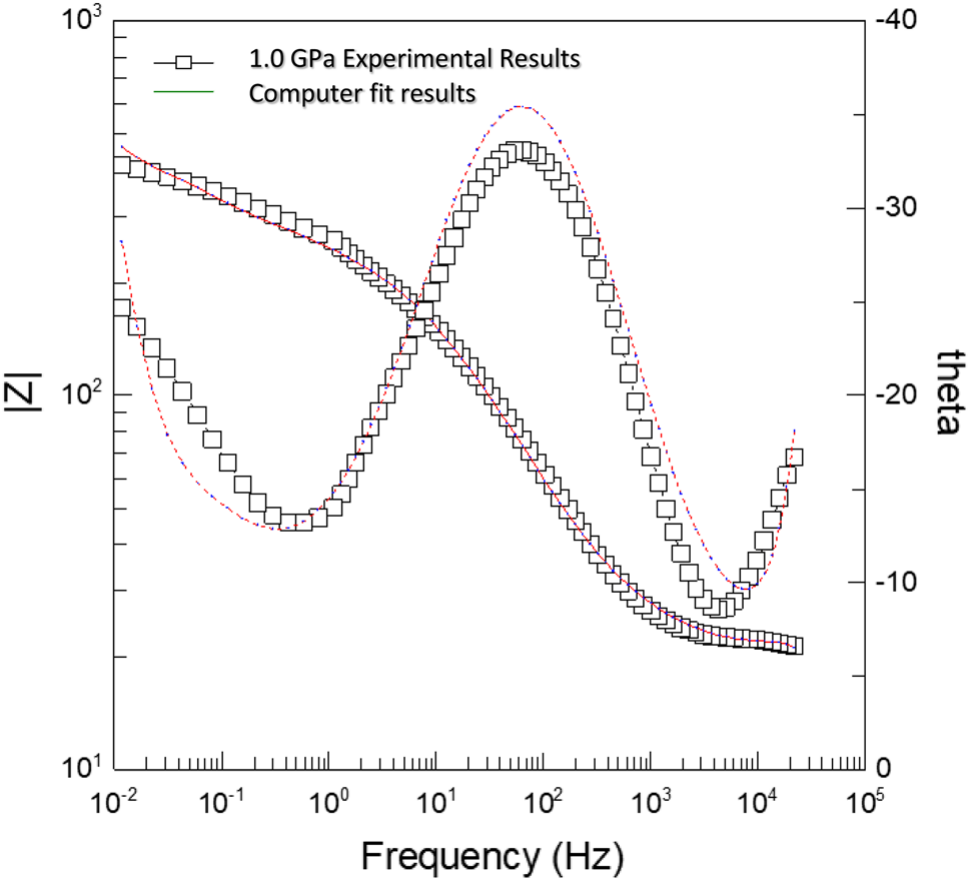
The experimental and fit result of Bode and theta plots for (Bi, Pb)-2223 superconductor electrode compacted at 1.4 GPa in 0.5 M HCl.

Figure 5 shows Bode and theta impedance plots of (Bi, Pb)-2223 superconductor electrodes compacted at 1.0 GPa in 0.5 M HCl. Theta plots show that the phase angle peak is less than 90, indicating non-ideal capacitive behavior of (Bi, Pb)-2223 superconductor electrodes that approve the system's inhomogeneities^19,20^.

**Figure 6:**
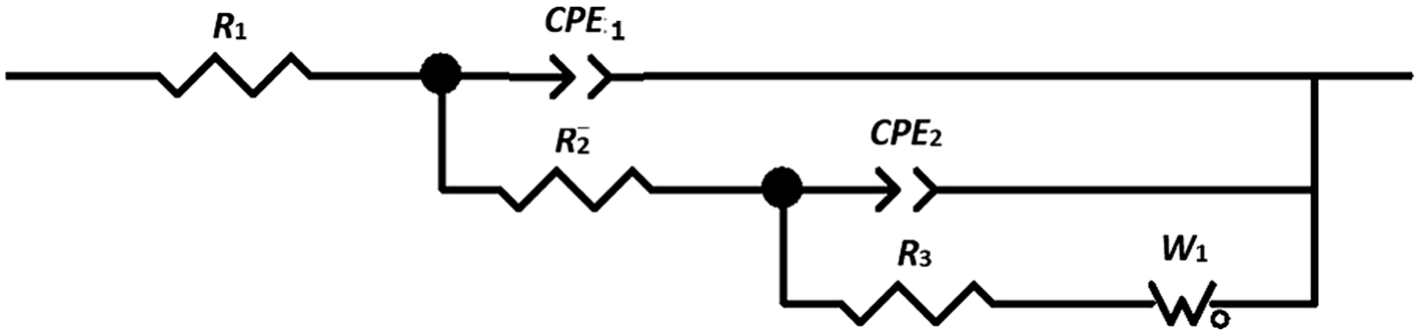
Equivalent circuit model used.

Figure 6 shows the equivalent circuit model used to analyze the curves to obtain the electrochemical impedance parameters. The elements of the used circuit are well described by Abdel-Gaber et al*.* in previous works^21^. The circuit is composed of several elements, including the solution resistance (*R*_1_), the resistance (*R*_2_) of the film formed on the surface of the superconductor surface, charge transfer resistance (*R*_3_), constant phase elements (*CPE*_*1*_ and *CPE*_2_), and the Warburge diffusion element (*W*_1_). The *CPE* consists of a none-ideal double layer capacitance (*Q*_dl_) and a constant (*n*). If *n* equals 1 then *Q*_dl_ is identical to that of a capacitor, *C*. When *n* is less than one, a depressed semi-circle is produced, and *Q*_dl_ represents a none-ideal capacitance. For the diffusion process, *n* equals 0.5 and a 45-degree line is produced on the Complex-Plane graph.

**Figure 7:**
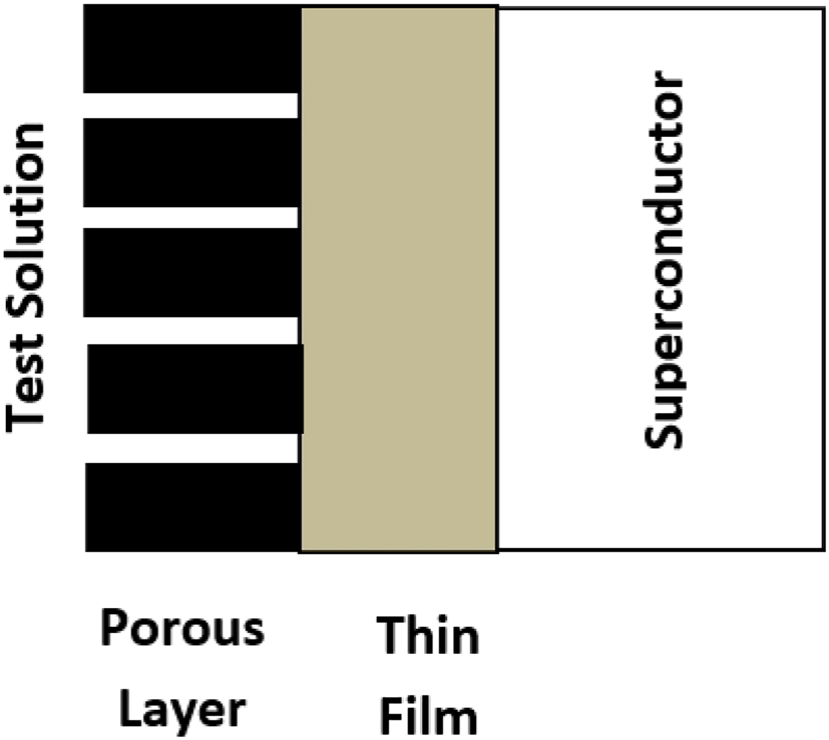
Schematic for physical interpretation.

This equivalent circuit is physically interpreted as the formation of two layers on the surface of the metal, one of which is porous to allow the aggressive ions to diffuse, Fig. 7.

Figure 5 indicates a reasonable fit of the experimental data to the used equivalent circuit. The electrochemical impedance parameters obtained from the analysis of the curves are shown in Table 4.

**Table 4:** The electrochemical impedance parameters of (Bi, Pb)-2223 superconductor electrodes compacted at different pressure in 0.5 M HCl.

Pressure GPa	R_s_ = R_1_ (ohm cm^2^)	Q_1_ (F cm^−2^)	R_2_ (ohm cm^2^)	Q_2_ (F cm^2^)	n_2_	R_ct_ (ohm cm^2^)	W_R_	W_T_	W_p_
0.3	6	6.8 × 10^–8^	13	4.0 × 10^–4^	0.70	32	420	98	0.35
0.7	10	6.9 × 10^–8^	9	3.9 × 10^–4^	0.68	54	523	50	0.48
1.0	14	6.9 × 10^–8^	7	2.5 × 10^–4^	0.67	229	542	41	0.49
1.4	17	7.0 × 10^–8^	6	2.3 × 10^–4^	0.67	267	571	38	0.49
1.9	19	7.1 × 10^–8^	4	2.1 × 10^–4^	0.67	135	794	4	0.49

The data indicated that the charge transfer resistance (*R*_3_ = *R*_ct_) increases with increasing compacting pressure up to 1.4 GPa, indicating a decrease in the corrosion rate. The decrease in the *R*_ct_ value at 1.9 GPa may be attributed to deformation and the formation of micro-cracks and defects because of the high compacting. The Warburg diffusion constant is described by three parameters: *W*_R_, *W*_T_ and *W*_P_. The *W*_T_ and *W*_R_ values represent the Warburg coefficient and Warburg resistance, respectively, and *W*_P_ is exponent which is set at 0.5 for finite length Warburg-short circuit terminus^22,23^. However, in finite length Warburg—open circuit terminus, 0 < *W*_P_ < 1. The obtained *W*_P_ values are in good agreement with the finite length Warburg—open circuit terminus. It is clear that the diffusion resistance (*W*_R_) increases with pressure. This can be explained using the Warburg coefficient parameter (*W*_T_) where in the diffusion interpretation, *W*_T_ = *L*^2^/*D*. (L is the effective diffusion thickness, and *D* is the effective diffusion coefficient of the particle)^20^. The data shows that increasing pressure decreases *W*_T_ and hence decreases the effective diffusion thickness and/or increases the effective diffusion coefficient.

**Figure 8:**
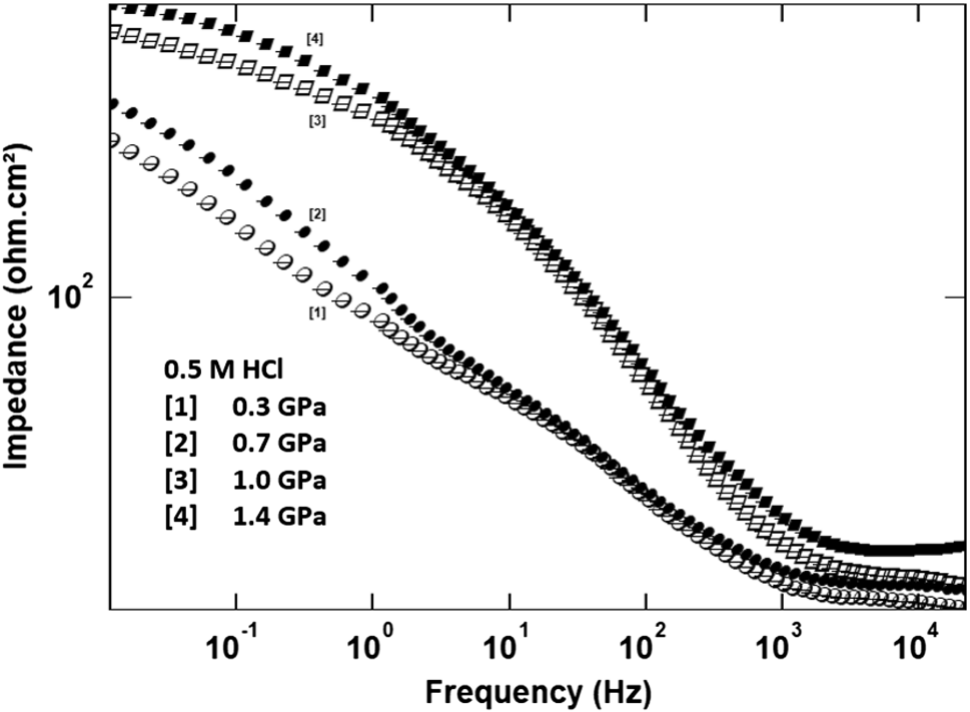
Bode impedance plots of (Bi, Pb)-2223 superconductor electrodes compacted at different pressure in 0.5 M HCl.

Figure 8 shows Bode impedance plots of (Bi, Pb)-2223 superconductor electrodes compacted at different pressure in 0.5 M HCl. The figure clearly demonstrates the presence of three-time constant. This is consistent with the used equivalent circuit, where the number of parallel RC or R-CPE components is commonly referred to as the number of time constants, which represent the time response when a signal is applied. Because the Warburg diffusion element is a component of (R-CPE) with constant n equal to 0.5. Therefore, there must be three-time constants. On the other hand, it is well known that the modulus impedance obtained at a minimum frequency, *R*_min_, could be related to corrosion resistance. Therefore, it is easily to predict that increasing pressure leads to increasing the resistivity of (Bi, Pb)-2223 superconductor electrodes.

**Figure 9:**
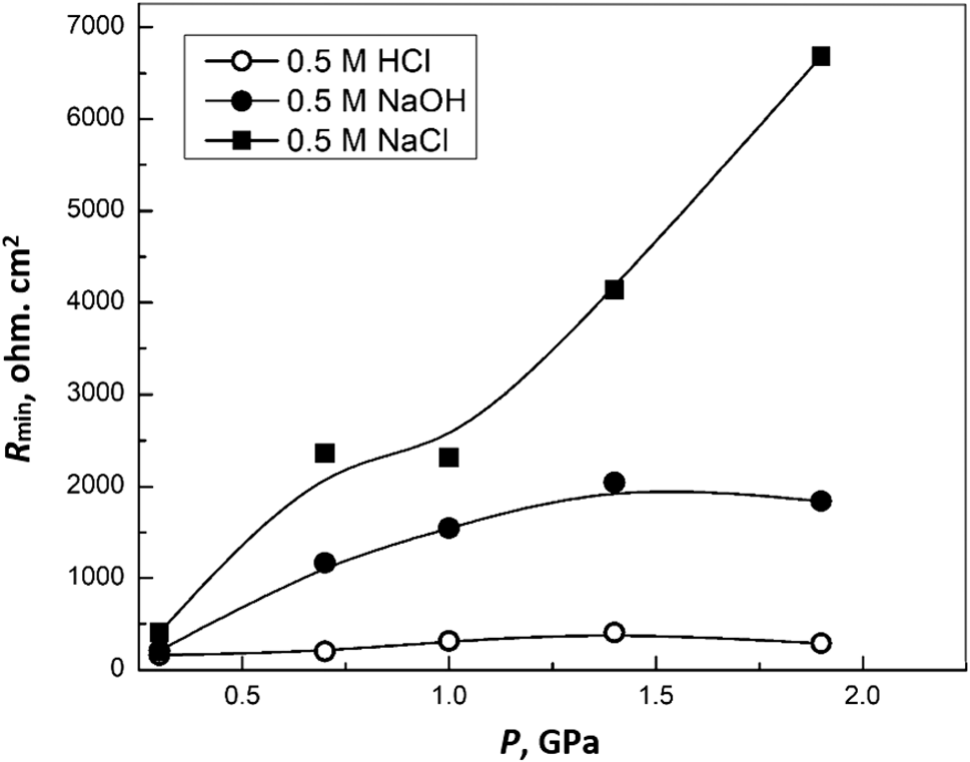
Variation of *R*_min_ for (Bi, Pb)-2223 superconductor electrodes compacted at different pressure in 0.5 M HCl, NaOH and NaCl.

Figure 9 shows the variation of modulus impedance at minimum frequency (*R*_min_), obtained from Bode impedance measurements for (Bi, Pb)-2223 superconductor electrodes compacted at different pressures in 0.5 M HCl, 0.5 M NaOH, and 0.5 M NaCl. As seen, the maximum protection (maximum *R*_min_) is obtained for the superconductor in 0.5 M NaCl, while the lowest protection is obtained in 0.5 M HCl. It is also observed that at 1.4 GPa, the maximum protection is acquired in the case of HCl and NaOH only. On the other hand, the *R*_min_ for the superconductor in 0.5 M NaCl varies exponentially with the pressure.

## Mechanism of corrosion

In the corrosion process, anodic and cathodic reactions occur simultaneously. The potentiodynamic polarization curves measurements of the (Bi, Pb)-2223 superconductor indicated that the cathodic reaction depends on the solution pH. The cathodic process in acidic solution (0.5 M HCl) is hydrogen evolution (2H^+^ + 2e^−^ → H_2_), whereas the cathodic process in alkaline or neutral solution (0.5 M NaOH, 0.5 M NaCl) is an oxygen reduction reaction (O_2_ + 2H_2_O + 4e^−^ → 4OH^−^).

As seen from Polarization and EIS measurements, the corrosion resistance of the (Bi, Pb)-2223 superconductor in NaCl > NaOH > HCl. These differences can be explained on the basis that the superconductor electrode may form a salt on its surface upon being attacked by an aggressive medium and that salt may or may not dissolve in the water of that medium. Peng Xing et al.^11^ reported the different ions and salts that exist during dissolution of the (Pb, Bi)-2223 superconductor at different pH values. They analyzed the chemical compositions of the deposits, leaching residue, and solutions by inductively coupled plasma atomic emission spectrometry (ICP-AES).

The anodic process of the superconductor could be explained by the formation of salt on its surface, which may or may not dissolve in the medium's water, as follows:

The (Pb, Bi)-2223 superconductor dissolves in HCl forming Pb^2+^ and Bi^3+^ that may explain its lower corrosion resistance.

1 $$\left(\mathrm{Pb},\mathrm{ Bi}\right){\text{-}}2223 \xrightarrow{\mathrm{HCl}} {\mathrm{Bi}}^{3+}+{\mathrm{Pb}}^{2+}$$

In the NaOH solution, it forms Pb(OH)_2_ and BiOOH salt.

2 $$\left(\mathrm{Pb},\mathrm{ Bi}\right){\text{-}}2223 \xrightarrow{\mathrm{NaOH}} \mathrm{BiOOH}+\mathrm{Pb}(\mathrm{OH}{)}_{2}$$

The difference in the corrosion rate depends on the solubility product constant (*K*_sp_) of the formed salt. Because Pb(OH)_2_ has a lower (*K*_sp_) (1.2 × 10^–15^) than BiOOH (4 × 10^–10^)^24^. As a result, it is possible to predict that BiOOH will be selectively dissolved from the super conductor in NaOH solution and that Pb(OH)_2_ salt will form on its surface.

In NaCl, the superconductor, on the other hand, forms Pb(OH)_2_ and BiOCl salt.

3 $$\left(\mathrm{Pb},\mathrm{ Bi}\right){\text{-}}2223 \xrightarrow{\mathrm{NaCl}} \mathrm{BiOCl}+\mathrm{Pb}(\mathrm{OH}{)}_{2}$$

BiOCl has a lower Ksp value (1.8 × 10^–31^) than Pb(OH)_2_, indicating that Pb is selectively leached in NaCl solution, resulting in the formation of the BiOCl salt at its surface.

Since, the solubility product constant Ksp denotes the extent to which a chemical can dissociate in water. The constant describes the tendency of a salt to develop on the superconductor surface, resulting in the formation of a film. It is well known that the formation of any film (even by adsorption) decreases the corrosion current.

Therefore, we can conclude the formation of a thick insulating layer in the case of NaCl leads to an increase in resistance by comparing the value of the Ksp of the BiOCl salt (1.8 × 10^–31^) formed on the surface of the superconductor in NaCl with the value of the Ksp of the Pb(OH)_2_ salt (1.2 × 10^–15^) formed in NaOH.

## Conclusions

- The corrosion rate of (Pb, Bi)-2223 superconductors depend on the compaction of the sample and the pH of the corrosive medium.
- The solubility of the various components of the superconductors in different media influences their resistance.
- The superconductor has a higher corrosion potential (*E*_corr_) in 0.5 M HCl than in 0.5 M NaCl or 0.5 M NaCl. This anodic shift reflects its higher tendency to corrode in 0.5 M HCl than the other tested corrosive media.
- Comparing the Ksp values for Pb(OH)_2_ salt formed over the super conductor surface in NaOH (1.2 × 10^–15^) and BiOOH salt formed over the surface in NaCl (1.8 × 10^–31^) indicates the formation of thick insulating surface film in NaCl clarifying that the corrosion resistance in NaCl must be greater than NaOH.
- Potentiodynamic polarization and EIS measurements indicated that the corrosion resistance of the (Bi, Pb)-2223 superconductor in NaCl > NaOH > HCl.

## Data Availability

The datasets used and/or analyzed during the current study are available from the corresponding author on reasonable request.
